# The make or buy debate: Considering the limitations of domestic production in Tanzania

**DOI:** 10.1186/1744-8603-8-20

**Published:** 2012-06-29

**Authors:** Kinsley Rose Wilson, Jillian Clare Kohler, Natalia Ovtcharenko

**Affiliations:** 1 Leslie Dan Faculty of Pharmacy, University of Toronto, 144 College Street, Toronto, M5S 3M2, Canada; 2 Associate Professor, Leslie Dan Faculty of Pharmacy and Munk School of Global Affairs, University of Toronto, 144 College Street, Toronto, M5S 3M2, Canada; 3 Research Assistant, Initiative for Drug Equity and Access, Leslie Dan Faculty of Pharmacy, University of Toronto, 144 College Street, Toronto, M5S 3M2, Canada

## Abstract

**Background:**

In order to ensure their population’s regular access to essential medicines, many least developed countries and developing countries are faced with the policy question of whether to import or manufacture drugs locally, in particular for life-saving antiretroviral medicines for HIV/AIDS patients. In order for domestic manufacturing to be viable and cost-effective, the local industry must be able to compete with international suppliers of medicines by producing sufficiently low cost ARVs.

**Methods:**

This paper considers the ‘make-or-buy’ dilemma by using Tanzania as a case study. Key informant interviews, event-driven observation, and purposive sampling of documents were used to evaluate the case study. The case study focused on Tanzania’s imitation technology transfer agreement to locally manufacture a first-line ARV (3TC + d4T + NVP), reverse engineering the ARV.

**Results:**

Tanzania is limited by weak political support for the use of TRIPS flexibilities, limited production capacity for ARVs and limited competitiveness in both domestic and regional markets. The Ministry of Health and Social Welfare encourages the use of flexibilities while others push for increased IP protection. Insufficient production capacity and lack of access to donor-financed tenders make it difficult to obtain economies of scale and provide competitive prices.

**Conclusions:**

Within the “make-or-buy” context, it was determined that there are significant limitations in domestic manufacturing for developing countries. The case study highlights the difficulty of governments to make use of economies of scale and produce low-cost medicines, attract technology transfer, and utilize the flexibilities of the WTO Agreement on Trade-Related Aspects of Intellectual Property Rights (TRIPS). The results demonstrate the importance of evaluating barriers to the use of TRIPS flexibilities and long-term planning across sectors in future technology transfer and manufacturing initiatives.

## Introduction

Global efforts to lower ARV prices and scale-up treatment access in Sub-Saharan Africa have fortunately resulted in price decreases from approximately US$10,000 per person per year (pppy) in 2000 to less than US$100 in 2007 [1]. Although treatment cost is not the only factor affecting access to medicines, it is obviously important. Access to ARV therapy in Tanzania has been steadily rising since 2004, with a coverage rate of 32% by 2010 guidelines (49% by 2009 guidelines) [2]. Still, the situation is not positive for all.

Treatment rates throughout Sub-Saharan Africa are still inadequate and, in 2007, 72% of HIV-infected individuals in need of treatment still remained without access to ARVs [3]. Considered alongside other notable barriers to treatment access, such as insufficient health care provision, poor political commitment, poverty, tariffs and taxes on imported medicines, drug access remains complex [4]. One major concern related to ARV access in developing countries is the World Trade Organization’s (WTO) Agreement on the Trade-Related Aspects of Intellectual Property (herein referred to as TRIPS) and the impact its patent terms have on ARV prices.

TRIPS came into effect in 1995 as a multilateral treaty that, for the first time, linked international trade liberalization with the protection of intellectual property (IP) including trademarks, copyrights, and patents. Under Article 33, TRIPS harmonizes the protection and enforcement of both pharmaceutical product and process patents, and therefore market exclusivity, for 20 years after their filing date (generally around the time of discovery). Harmonization sets a universal standard for patenting; if countries do not enforce agreed international standards, they are potentially subject to sanctions imposed by the WTO.

One strategy that developing countries are encouraged to use for price reduction is the domestic manufacture of drugs. The logic for this strategy stems from dependency theory and import substitution. The focus shifted to regulation and effective distribution after the development of the WHO Essential Medicines Programme in 1975, but the possible role of domestic manufacturing was revisited with the onset of the TRIPS Agreement and the subsequent public health focus in the Doha Declaration in 2001.

Article 7 addresses the transfer of technology and development of technological capabilities that should result from IP protection. It requires, “the promotion of technological innovation and to the transfer and dissemination of technology” [5]. Article 66.2 in particular encouraged developed countries to provide industry incentives for pharmaceutical technology transfer and capacity building in developing countries [5]. Provisions outlined in the TRIPS Agreement and the Doha Declaration of 2001 prioritize public health over trade and allow developing countries to manufacture patented medicines in cases of national emergency. The Doha Declaration clarified the criteria of national emergency to clearly include public health crises [6]. The August 2003 Decision then provided a mechanism by which countries without manufacturing capacity could utilize the compulsory licensing flexibility in light of the TRIPS requirement that it be used for domestic market only [7]. This provision allowed countries with manufacturing capacity to file for a compulsory license for the purpose of export – however this flexibility has only been used once since its implementation.

Patent protection under the TRIPS Agreement imposes limits on when and how local production can take place. Nevertheless, some developing countries that are obliged to implement TRIPS by 2005 have managed to navigate its restrictions at times to produce drugs locally. Strategies have included manufacturing drugs through voluntary licenses with pharmaceutical companies and compulsory licenses for patented products. Some examples of compulsory licensing are Zimbabwe in 2002 for a selection of ARVs (including stavudine, nevirapine, and zidovudine), Brazil in 2007 for efavirenz, Ecuador in 2010 for ritonavir [8][9][10]. The Doha Declaration extended the transition period for LDCs to 2016 resulting in less restricted access to generic versions of drugs. Recently, there has been discussion of extending this transition period further, a move supported by the International Federation of Pharmaceutical Manufacturers & Associations [9]. But as this case study will show, barriers remain to improving access through technology transfer in these countries.

Least developing and developing countries need to consider whether it is in their best interest to produce drugs domestically or import them from existing generic producers, such as those in India. The make or buy dilemma weighs which strategy would be most cost effective considering the low prices that must be matched by domestic manufacturers. There is compelling evidence on both sides; some research presents a case for affordable drug production in developing countries [11][12], while others argue that it makes little sense economically, as such initiatives are most often not reliable and do not reduce prices [13][11][14]. On the ‘Buy-Side’, affordability is a key concern. There are a number of measures developing country governments can utilize to increase the affordability of imported ARVs. These include generic competition, negotiation with patent holders and bulk procurement. On the ‘Make-Side,’ public health interests, economic interests and technological developments (i.e. manufacturing capacity) should be addressed.

Domestic manufacturing also does not guarantee greater stability in supply, a key component of access to medicines. Despite this, there are compelling arguments that it brings benefits in addition to price reduction. Domestic manufacturing keeps money in the economy, by employing people and investing in infrastructure and facilities. Backing this, the African Union [15] argues that domestic drug production develops the appropriate industrial and technical infrastructure that can enhance long-term health security, self-sufficiency, employment, foreign exchange, in addition to access to essential medicines. In other words, local manufacturing potentially can bring economic and symbolic gains to a country.

We examine the “make or buy” dilemma and the critical limitations of domestic manufacturing through the case study of Tanzania’s attempt to domestically manufacture ARVs with an imitation technology transfer agreement. Tanzania is a least developed country (LDC) according to United Nations criteria [16], with 35.7% of the population living below the poverty line and a GNI of US$500 per capita [17]. There are 1,400,000 people living with HIV only 32% of which are undergoing treatment [2]. With a commitment to increase ARV availability, the government considered domestic production as a means to scale up treatment to improve the health of its population. The local manufacturer Tanzania Pharmaceutical Industries (TPI) entered an agreement to produce a triple FDC 17 of first-line ARVs lamivudine, stavudine, and nevirapine (3TC + d4T + NVP), named TT-Vir.

This paper examines several barriers that TPI faces in establishing secure local manufacturing of ARVs. The limited support from policy makers, the conditions of the technology transfer agreement, its level of manufacturing capacity, and its ability to compete in domestic and regional markets are considered. These factors suggest that the limits to affordability in TPI’s ARV manufacturing strategy are not overcome by the economic interests of the government. There is no strong commitment from the Tanzanian government on the use of TRIPS flexibilities; instead ministries are pressuring for increased IP protection. These considerations weaken the ‘Make’ side of the debate. On the ‘Buy’ side, the case study suggests that TPI will not be able to match donor prices (within 10-15% to ensure affordability) [18]. As well, TPI’s limited manufacturing capacity, its lack of WHO prequalification status and the difficulty of entering broader markets hurt TPI’s ability to price its ARVs competitively. As a result of these barriers, TPI cannot meet the necessary economies of scale and offer competitive prices.

## Methods

Purposive sampling of documents, key informant interviews and event driven observation were used to collect data for this case study. A broad range of documents were collected, including reports from international organizations (World Bank, WHO, WTO), government publications, and local pharmaceutical industry documents. Document collection began with a search of publicly accessible documents and was supported by recommendations from key informants.

### Selection of key documents analyzed for data collection

· Patent legislation

· Health legislation

· National drug policies

· Science and technology policies

· Industrial policies

· National AIDS plans

· ARV tender documents

· Bilateral/multilateral/international trade agreements

· Submissions to government by interest groups

· Local pharmaceutical industry assessments

· Annual reports of drug firms

· Newspaper articles, industry newswires, and press releases

A total of 24 semi-structured interviews were conducted in Tanzania (by KRW) over a three -month period. Representatives from government agencies (9 interviews), the pharmaceutical industry (11 interviews), and international, bilateral organizations and NGOs (4 interviews) were interviewed. A series of open ended questions were used in all interviews which prompted new questions based on responses. Interviews progressed from a broad to more explicit conversation.

### Organizations interviewed for data collection

#### Government representatives and agencies (9 interviews)

· Ministry of Health and Social Welfare

· National AIDS Control Programme

· Tanzania Food and Drug Administration

· Medical Stores Department

· Commission for Science and Technology

· Tanzania Investment Centre

· Business Registration and Licensing Agency

#### International, bilateral organizations and NGOs (4 interviews)

· WHO

· PEPFAR

· UNIDO

· Action Medeor

#### Pharmaceutical industry (11 interviews)

· Tanzania Pharmaceutical Industries

· Local drug firms and subsidiaries

· Local distributors

· Technology suppliers

### Semi-structured interview guide

#### Background

What is the current context in your country with regard to HIV/AIDS and ARV drug access?

What led to the decision to locally produce patented ARVs?

#### Factors influencing technology transfer arrangements

What led to the development of the current TT arrangement?

What are the most important factors that shaped the transfer arrangement?

How have national policies in place affected the development of the transfer arrangements?

How have international policies affected the development of the transfer arrangements?

How has the manufacturing and technological capacity of the recipient firm affected the development of the transfer arrangements?

How would you compare your country’s situation with those of others in your region?

#### Process of technology transfer

How much communication is there between supplier and recipients firms?

What are the terms or conditions of the transfer arrangement?

What is/was the duration of the transfer arrangement?

What have been the processes involved in the transfer of technology?

What type of knowledge was/is exchanged during the transfer (for example, technical, informational, managerial, training, etc.)?

What are the end goals of the transfer arrangement

What was the greatest obstacle in the transfer arrangement?

What are the most important elements of effective technology transfer?

How would you compare this arrangement with other possible technology transfer arrangements in your region?

#### Contextual

How would you define technology?

How would you define technology transfer?

Purposive sampling also assured that those interviewed and observed had specific knowledge on the events leading to technology transfer arrangements and local manufacturing initiatives. Data collection continued until evidence collected did not produce new information or themes [19]. Interviews began with open-ended questions and progressed to a tighter structure.

Participant observation was used to gather evidence and to gain an in-depth understanding of the processes surrounding the technology transfer arrangement. One week was spent at TPI and a High Level Meeting on Intellectual Property was attended. Observations were guided by the semi-structured interview questions and were recorded based on what was observed, who was observed, and when and where the sample was observed [20].

Open coding was used to identify themes in the documents, interviews, and observations. Preliminary coding of interview transcripts was done by two coders (KRW and JCK). Data was then grouped into thematic categories and relationships were identified and more closely analyzed.

### Key concepts

The transfer of technology is an unclear term, particularly in terms of its implications under the TRIPS Agreement, as it allows for various interpretations. The definition of *technology transfer* for the purposes of this research integrates the definitions by Maskus [21] and UNCTAD [22]. It is understood as a process for the transformation of information between a technology supplier and a recipient for the manufacture of ARVs. Technology transfer can range from the exchange of technical knowledge through formal documentation, such as a license to exploit a patent, or through technical know-how and assistance in reverse engineering an imitation of the product. Significantly, there must be intent to pass on technological information from a supplier to an unrelated recipient firm. For example, in this research, FDI for subsidiaries in developing countries is not considered transfer of technology. This is because the patent holder remains the only right holder of the information, and the effective monopoly conferred by the patent status stays intact.

This case study considers an *imitator* (reverse engineering) technology transfer arrangement An imitator arrangement occurs when the supplier is not the patent holder, but a generic firm or any other type of organization with the knowledge to formulate and. In this case, the development of manufacturing capacity through the construction of a new ARV manufacturing facility facilitates imitation. ARV manufacturing capacity is the ability of a local firm to develop a number of quality ARVs and produce them at a competitive international price. These arrangements rely on individuals and organizations external to the patent holders that have knowledge on the production of the patented ARV and commonly include more extensive technology transfer terms than found in voluntary licenses.

Within the framework of the make or buy debate we focus heavily on technological capabilities on the make side and generic competition on the buy side. The level of technological capabilities in developing countries has been described by a number of sources [23][24][18][25-27]). Three levels of production are usually delineated: primary, secondary, and tertiary production. Primary production, most prominently found in India and China, includes the conversion of raw materials and intermediates to active pharmaceutical ingredients (APIs). Secondary production involves the processing and formulation of finished dosage forms from APIs, and is found in countries such as Brazil and South Africa (although facilities in both countries have some API production as well). Tertiary production packages and labels finish products from primary and secondary sources. Generally, countries first develop the capacity for tertiary production, which can help build the requisite skills and experience for higher levels of production over time [13].

Generic competition commonly used as a mechanism for driving down drug prices, having a significant impact on access to medicines. It has had a polarizing effect on the make-or-buy debate due to the rapid growth of the Indian generic industry. As India did not have to enforce product patents until after the 2005 transition period it expanded its generic industry with the help of advocates and international organizations such as the William J. Clinton Foundation and MSF. By 2007, the Clinton HIV/AIDS Initiative (CHAI) negotiated to drop the price of the first-line treatment tenofovir + emtricitabine + efavirenz (TDF + FTC + EFV) from the 2007 price of US$487 pppy (MSF, 2007) to US$349pppy in 2008 [28][1]. This was a huge change from the lowest price in 2000, US$10,439 pppy [1].

## Results

### Policy background

Tanzania lacks a coherent policy strategy for the development of its industry. Moreover, pharmaceutical patent enforcement is weak and investment in science and technology is stagnant, a scenario common in many developing countries. This provides little incentive for industry to invest in local manufacturing. Tanzania’s technology transfer agreement was undertaken by a partly state owned company without WHO prequalification of its drugs, another disincentive for private investors.

The impetus for the technology transfer for the production of the first-line treatment 3TC + d4T + NVP came from Tanzania’s need to drastically scale-up HIV treatment to match its 2008 goal of treating 423,000 HIV-infected individuals with ARVs [29]. By 2006 only 64,000 individuals had been treated out of 1.4 million HIV-infected citizens [30]. Enhancing research capacity, research infrastructure, and technology transfer are key priorities for Tanzanian policy and are necessary for growth in the pharmaceutical sector. Even so, there is little evidence of solid commitment [31].

Shortfalls in support extend to local drug production and the use of TRIPS flexibilities. We found that only the Ministry of Health and Social Welfare (MoHSW) explicitly promotes the local pharmaceutical industry. In contrast, the Ministry of Trade and Industries and the Ministry of Science and Technology emphasize increasing IP protection. Although Tanzania’s 1992–2000 Pharmaceutical Master Plan (PMP) committed to the objective of increasing the procurement of locally manufactured drugs from 30% to 60% of market share by 2010 the tender process has been under scrutiny for lack of transparency and possible corruption [32].

The level of enforcement of patent protection in Tanzania is an important determinant of the success of local drug manufacturing. Tanzania has until 2016 to comply with TRIPS, but terms Articles 7(1) and 8 of the 1987 Tanzanian Patent Act make both pharmaceutical process and product patents available [33]. Article 38(1) of the Tanzanian Patent Act states that patents are only granted for 10 years from filing, as opposed to the 20 years mandated by TRIPS. Yet the information published on the website of the Business Registrations and Licensing Agency states that a 20 year patent term is given [34]. However, the strength of the enforcement surrounding the patent law is unclear. Informants assumed that patents were not enforced by the government or industry. This scenario is not atypical in Sub- Saharan Africa where patent holders have waived their IP rights for many first-line ARVs, with the exception of patents held in South Africa [35]. Information on the current patent policies is also inconsistent.

Two of the five first-line ARVs included in the FDC treatment manufactured by TPI, 3TC and NVP, were patented in Tanzania but both expired in 2001 under the 10 year patent term. Consequently, the manufacturing of this ARV does not contravene the Tanzania Patent Act or the TRIPS Agreement. Though Tanzania can be seen as exercising the 2016 transition period, the use of the TRIPS flexibilities is not part of a legislative framework. Although the MoHSW is in support of such a change, other agencies aim to enact change in the other direction.

### Technology transfer arrangement

Though originally state-owned, TPI was partially privatized in 1997. Currently, 40% of its shares are held by the government and 60% by Tanzanian entrepreneurs. The WHO does not promote government-led local production, encouraging them to focus on regulatory mechanisms [15]. The perception of the capacity of state-owned pharmaceutical firms, however, varied across informants. Some pointed to a lack of transparency, corruption, poor management, and bureaucracy that create inefficiencies within the industry. In fact, the *Global Competitiveness Report 2008/2009* named corruption as one of the most problematic factors for doing business in Tanzania [36]. This may deter private investors who believe that a government share in TPI increases bureaucratic procedures and reduces transparency.

Still others expressed a positive view of government-sponsored industry. Within its grant application to the European Commission, Action Medeor emphasized the sustainability of the technology transfer initiative given TPI’s status as an enterprise with government shareholders that support the project as part of its responsibility and operations. An informant at the European Commission acknowledged that more government involvement and support may have enhanced TPI’s grant application as it assured the country’s commitment to address HIV/AIDS and improve sustainability of supply. Although, as mentioned earlier, domestic manufacturing does not necessarily lead to greater sustainability.

TPI’s manufacturing capacity for bulk pharmaceuticals prior to the European Commission grant was limited [18][25][27]. TPI does not have API manufacturing capacity; a 5-year fixed cost agreement with China’s MChem maintains its supply. It has some secondary production capacity, making primarily antibiotics, analgesics, and antimalarials with recent expansion to artemisinin-based antimalarials following privatization and another technology transfer agreement.

The arrangement under study began in October 2005 between TPI and a former member of the Research & Development Institute of the Government Pharmaceutical Organization (GPO) of the Ministry of Health, Thailand. In November 2006, a formal partnership was established with German non-governmental organization (NGO), Action Medeor, to construct a new ARV manufacturing facility financed by a 48-month, US$6 million grant from the European Commission. Two primary goals of the grant were to build a facility in line with WHO prequalification standards and to build technical capacity to produce ARVs.

The grant had some limitations which led to some concern amongst informants. It did not cover the cost of the prequalification process itself or the cost for developing drugs. Industry informants estimated the cost of a single oral dose of the ARV to be between US$800,000 and US$1 million. There were also doubts that the US$6 million grant would cover the cost of the new manufacturing facility. A similar partnership between the Indian firm Cipla and Ugandan firm Quality Chemicals had the Ugandan firm raising $38 million internally to construct an internationally certified facility [37]. Finally, informants were not convinced that the grant was sufficient to produce a facility compliant with WHO Good Manufacturing Practices (GMP). Nevertheless, the facility was completed in late 2011. Additional contributions from TPI, which had not been confirmed at the time of interviews, were made. These consisted of US$963,000. Action Medeor also gave US$600,000 more [38].

### Competitiveness

The competitiveness of the first-line ARV market means TPI needs sufficient economies of scale along with WHO prequalified ARVs to meet the target price range. Like many Sub-Saharan African and LDC manufacturers, TPI only has limited capability to formulate and package bulk pharmaceuticals, limiting its ability to compete [39][25][27].

Production scale is a significant disadvantage for an LDC manufacturer when attempting to reach economies of scale. The facility funded by the European Commission is designed to manufacture 100 million units per year. This is in contrast to the existing 500 million tablets per year capability for Tanzanian firms and 1.2 billion tablets per year for South African and Indian firms [40]. Local manufacturers also face additional costs which are not as great a concern for multinational generic firms. Because they do not have primary production capacity, the cost of importing APIs must be considered. These play a much smaller role for integrated generic manufacturers. Packaging material is another supplementary cost. Though no taxes are paid for API or intermediates imports, they are paid for the packaging materials. This can result in higher taxes for local firms than for importers [40]. Import costs (freight costs) themselves, often considered a strong area of savings for local firms, only accounts for 4% of the 25% difference in Tanzanian and Indian generic firm production costs [40].

Large tenders are also needed to obtain economies of scale. In this regard, donor financed drugs in Sub-Saharan Africa present the biggest barrier for local manufacturers in Tanzania. Many donors, including the Global Fund for HIV/AIDS, Tuberculosis and Malaria and PEPFAR focus on ARV procurement but require compliance with international quality standards. National tenders financed by the government exist but the large number of people needing treatment and the availability of donor financing mean that the government generally does not fund ARV programs entirely.

TPI has not been granted certification by the WHO Prequalification Programme or approval by the United States Food and Drug Administration (participation in PEPFAR tenders), thereby disqualifying the firm from competing in donor-financed ARV tenders. WHO Prequalification vouches for the safety and efficacy of a company’s products. To obtain it a company must first get an invitation from the WHO to submit a product for evaluation, submit data on the quality, safety, and efficacy of the product, and have its manufacturing sites assessed [41]. It can take from 3 to 24 months and cost a firm up to $200,000. Technology suppliers suggested that TPI could potentially avoid the barriers of WHO prequalification and international competitive bidding by producing off-patent drugs for opportunistic infections related to HIV/AIDS. But although technology suppliers have indicated there is a market demand for domestic production of these drugs no interest has been demonstrated by the company.

Even in situations where TPI can enter the market (i.e. government tenders), there is a vast price differential in the products manufactured by TPI and its cheaper competitors. In January 2007, TPI estimated to manufacture 3TC + d4T + NVP for US$240 pppy while Cipla’s prequalified FDC went down to US$92 pppy with competition [42]. The MSD allows 15% equalization for domestic competitors, but price competition with multinational generic firms is still a concern. Economies of scale are a key factor in the lower prices of large firms, but local industry informants also suggested that predatory prices are used to prevent small firms from entering the market.

Competition concerns also extend beyond large foreign manufacturers; domestic competition is an increasing concern. The School of Pharmacy at the Muhimbili University College of Health Science is assessing the possibility of opening a pharmaceutical plant for training pharmacists and produce medicines. In addition, the local firm Shelys Pharmaceuticals was acquired by Aspen Pharmacare, the largest pharmaceutical manufacturer in Sub-Saharan Africa. In early 2008, Shelys Pharmaceuticals entered into a technology transfer agreement with Roche to strengthen their capacity in ARV manufacturing [43]. These firms would also receive the 15% equalization preference given to domestic manufacturers in tenders. The August 30 Decision allows export of generics to other countries in regions where over 50% have LDC status. Although TPI aims to expand into EAC markets and the South African Development Community, its inability to compete effectively in the domestic market suggests it will be more challenging for TPI to participate in the regional markets. Regional exports from Tanzanian companies generally pay the same import tariffs as exports from Asia with no advantage gained from Sub-Saharan African trade agreements [40]. Labour costs are likely lower in Tanzania, but when compared to other countries with generic manufacturing initiatives (such as India, China, Kenya and South Africa), they relate poorly to worker productivity [36]. Furthermore, because of the prominence of donor-financed tenders throughout Sub-Saharan Africa, the lack of WHO Prequalification would continue to be a limitation.

## Discussion

The “make-or-buy” debate, in the context of ARVs in Tanzania, exemplifies the challenges local pharmaceutical manufacturers face in least developing countries in an effort to ensure better access of the population to essential medicines. The barriers to local firms are great and we found that there is consequently a lack of strong incentives for international firms to transfer technology for the purpose of domestic ARV production in Tanzania. Several key concerns emerged in the analysis:

· **Lack of political support**: there is no consensus amongst different ministry agencies on the appropriate level of IP protection. Although the technology transfer agreement examined here can be interpreted as a use of the TRIPS flexibility for LDC countries, it was not put in place due to any legislative framework in Tanzania.

· **Limited production capacity:** although informants suggested that TPI would be more successful if it entered the market for other classes of drugs such as those for opportunistic infections related to HIV/AIDS, this is not the direction which the company chose to take. Yet its capacity for generating economies of scale for ARVs through savings in production is limited making it difficult to price products competitively.

· **Limited competitiveness in multiple markets:** TPI is greatly limited by its lack of WHO prequalification which excludes it from donor financed tenders. It must also face emerging domestic competition and significant cost barriers in entering regional markets.

By assessing the domestic conditions affecting the development and affordability of locally manufactured ARVs in Tanzania, the case study results are useful to developing country governments faced with securing low-cost essential medicines for their populations as well as building their countries’ technological capacity. Within the “make-or-buy” context, this paper highlights the difficulty of domestic manufacturers to supply affordable medicines when a large number of high volume international competitors exist. It suggests that LDCs, with little financing, have limited opportunities to attract the transfer of technology, regardless of their IP status.

Lessons learned from the study include:

· Cohen-Kohler’s [44] suggestion that even though TRIPS provisions offer governments the ability to put health before trade objectives, many developing countries and LDCs simply cannot utilize these provisions is reinforced.

· Barriers in administration, politics, policy, and capacity limit the use of TRIPS flexibilities in LDCs.

· Successful technology transfer arrangements and manufacturing initiatives require long-term coordination and planning across health, industrial, and science and technology sectors.

· Efforts to develop industry will have great difficulty in succeeding if driven entirely by the health sector

· The need to address adequate economies of scale of [QA = quality assured?] production must be established from the start in the initial business plan

These factors should be addressed upfront by developing country governments and firms, along with other noted barriers, to evaluate whether or not the initiative is viable. Detailed cross-sector planning is imperative for developing countries to provide sustainable drug supplies for their populations in an effort to improve population health outcomes.

## Competing interests

The authors declare that they have no competing interests.

## Authors’ contributions

KRW designed the theoretical framework, performed field work, and data collection and analysis. JCK participated in the theoretical framework design, data analysis, manuscript drafting and revision. NO participated in manuscript drafting and revision. JCK and NO read and approved the final manuscript. This work is based on the thesis written by KRW under the supervision of JCK, KW did not contribute to the article submission process.
